# Observed electric charge of insect swarms and their contribution to atmospheric electricity

**DOI:** 10.1016/j.isci.2022.105241

**Published:** 2022-10-24

**Authors:** Ellard R. Hunting, Liam J. O’Reilly, R. Giles Harrison, Konstantine Manser, Sam J. England, Beth H. Harris, Daniel Robert

**Affiliations:** 1 School of Biological Sciences, University of Bristol, Bristol, UK; 2 Department of Meteorology, University of Reading, Reading, UK

**Keywords:** Atmospheric science, Atmosphere modeling, Entomology

## Abstract

The atmosphere hosts multiple sources of electric charge that influence critical processes such as the aggregation of droplets and the removal of dust and aerosols. This is evident in the variability of the atmospheric electric field. Whereas these electric fields are known to respond to physical and geological processes, the effect of biotic sources of charge has not hitherto been considered. Here, we combine theoretical and empirical evidence to demonstrate that honeybee swarms directly contribute to atmospheric electricity, in proportion to the swarm density. We provide a quantitative assessment of this finding, by comparing the electrical contribution of various swarming insect species with common abiotic sources of charge. This reveals that the charge contribution of some insect swarms will be comparable with that of meteorologically induced variations. The observed transport of charge by insects therefore demonstrates an unexplored role of biogenic space charge for physical and ecological processes in the atmosphere.

## Highlights

• Aerial insects carry an electric charge
• Swarming honeybees have a density-dependent effect on atmospheric electricity
• Insects can have similar effects on atmospheric electricity as weather events

## Graphical abstract

**Figure:**
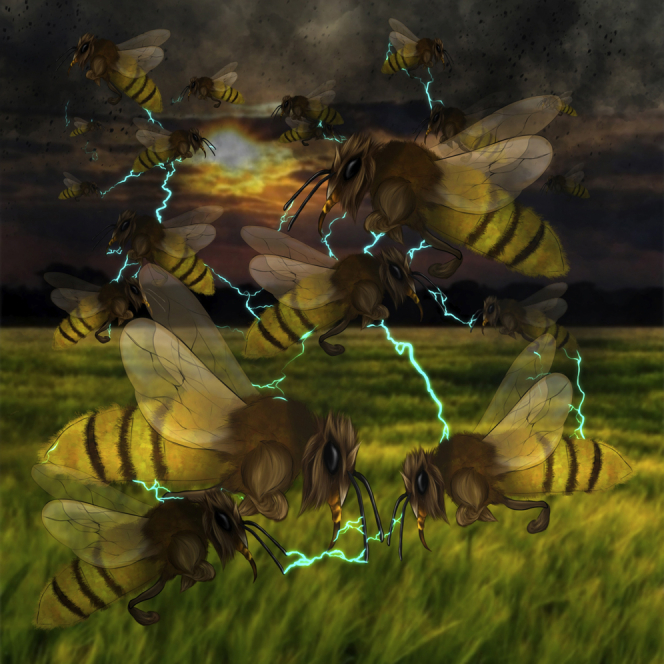

## Introduction

The Earth’s atmosphere is always electrified to a greater or lesser extent, even in fair weather away from thunderstorms. Fossil evidence of paleolightning (Harland and Hacker, 1966) indicates this is unlikely to be a new phenomenon geologically, and therefore the atmospheric electric field can be regarded as a fundamental atmospheric property. It is conventionally observed as the vertical Potential Gradient (PG), essentially the voltage difference between the Earth’s surface and a point (often 1 m) above it (Everett, 1868; Fdez-Arroyabe et al., 2021; Whipple, 1929). The atmospheric PG has proved important in understanding large-scale drivers of the global atmospheric electric circuit (e.g. from climate and space weather) (Harrison, 2015), and in causing migration of biologically relevant ions (e.g., nitrate, sulphate, radon) (Hunting et al., 2019, 2021a, 2021b) as well as facilitating animal dispersal or navigation (Clarke et al., 2013; Hunting et al., 2022; England and Robert, 2022; Morley and Robert, 2018). The continuous monitoring of atmospheric electric fields is therefore essential in developing a detailed understanding of a wide array of atmospheric, biological, and geological processes.

Various drivers of variations in atmospheric electricity have been identified. On a large scale, PGs in fair-weather regions are a consequence of global electric current flows driven by thunderstorm regions (e.g., Haldoupis et al., 2017; Rycroft et al., 2000). On a local scale, the PG is influenced by local atmospheric electrical processes such as cloud electrification, lightning initiation, precipitation, aerosol charging, and radioactivity (Anisimov et al., 2017; Hamilton, 1965; Harrison and Aplin, 2002; Pierce, 1972; Takeda et al., 2011), and is sensitive to anthropogenic pollution (Matthews et al., 2019), volcanism and potentially earthquakes (Harrison et al., 2010; Kamogawa et al., 2004). Yet, while these various abiotic drivers have been identified, they can still fail to explain commonly observed variations in atmospheric PGs, suggesting a comprehensive understanding of the causes and consequences of spatio-temporal variability in atmospheric electricity is still incomplete.

We propose a new perspective based on the idea that organisms that inhabit the lower atmosphere can act as a source of atmospheric space charge and associated atmospheric electrical variability. Insects are well-known to be ubiquitous in the global atmosphere (Hu et al., 2016; Reynolds et al., 2017), and they can occur in large densities in the lower (∼0–5 km altitude) atmosphere. Moreover, a wide array of airborne insect species have been shown to carry an electric charge, which ranges from picocoulombs to nanocoulombs per individual (Edwards, 1962; England and Robert, 2022; Erickson, 1975). These observations support the hypothesis that large aggregations of aerial insects provide an important source of space charge in the atmosphere. Here, a honeybee swarm is investigated as an emblematic example of such an insect aggregation, as honeybees have predictable swarming behavior, and the existence of individual bee charges has been well documented. We provide model-based and empirical evidence that bee swarms make a density-dependent contribution to the atmospheric PG, and explore the broader significance of insect swarms by offering a comparison with known abiotic sources of atmospheric electricity.

## Results and discussion

### Effect of honeybee swarms on atmospheric electricity

**Figure 1:**
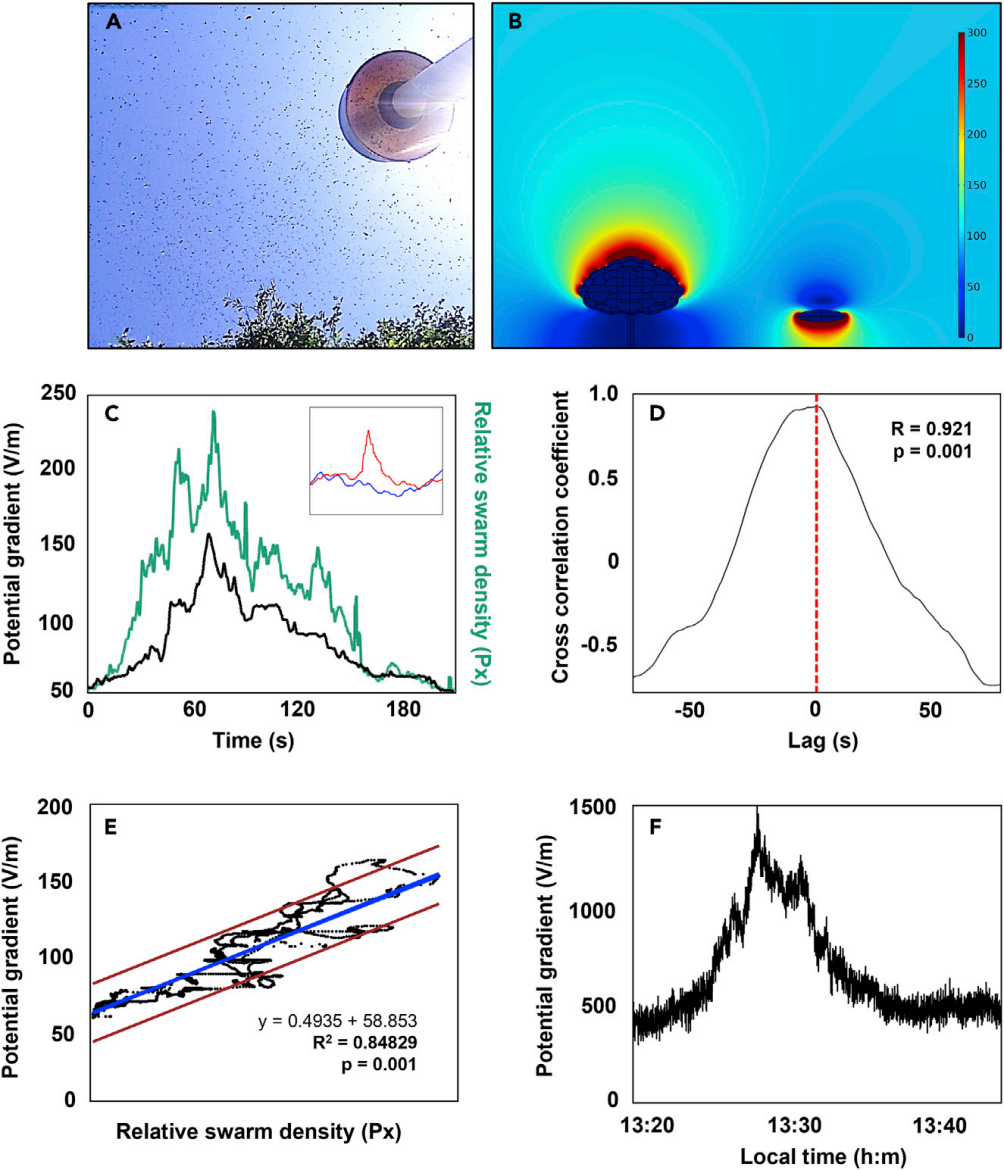
Effect of honeybee swarms on the atmospheric potential gradient, PG (A) Honeybees passing the electric field monitor at the experimental site. (B) Finite element model illustrating the potential effect of a honeybee swarm on atmospheric PG (in V/m). Color scale truncated above 300 V/m. (C) Atmospheric PG and honeybee swarm density (expressed as the pixel density, Px. Inset shows dynamics in PG for both the honeybee swarm (red) and open field conditions (blue). (D) Cross-correlation analysis between atmospheric PG and honeybee swarm density. (E) Linear regression analysis between atmospheric PG and honeybee swarm density. (F) Changes in atmospheric PG in response to another honeybee swarming event.

**Figure 2:**
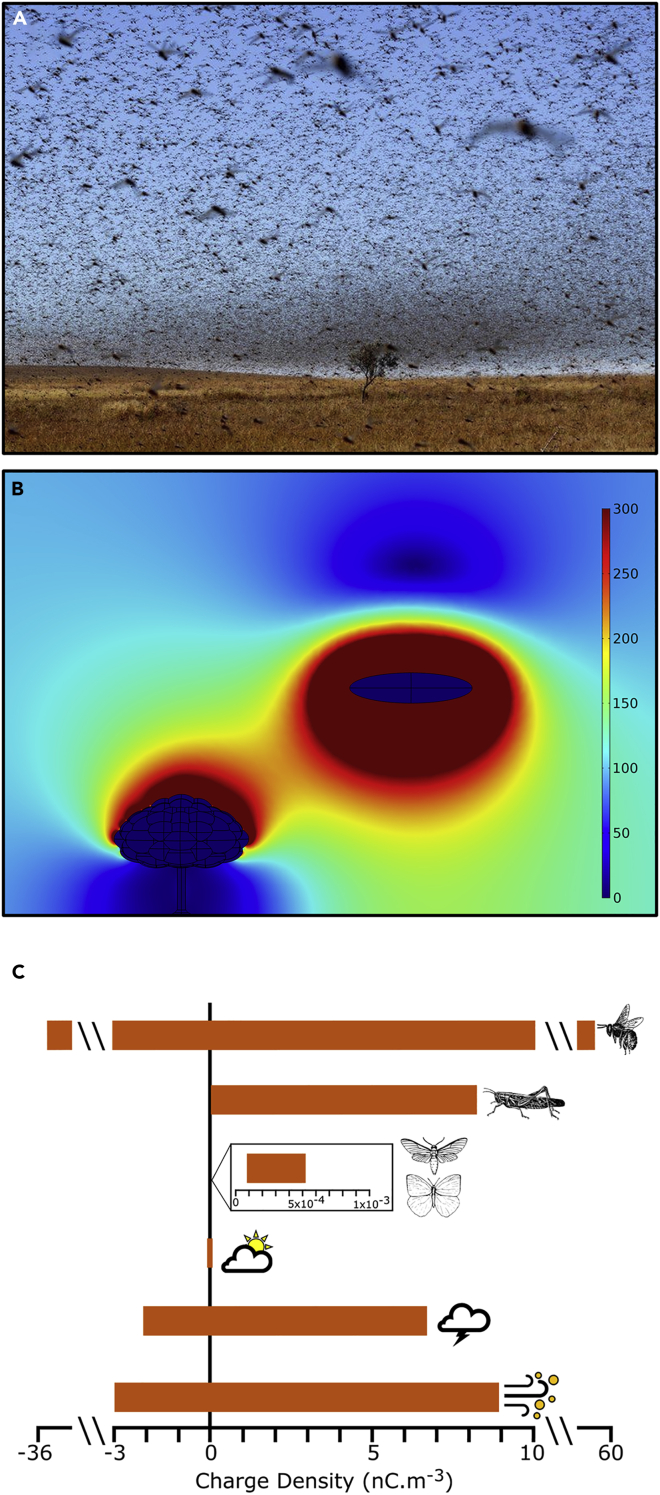
Effect of a locust swarm on the atmospheric potential gradient and the significance of insect swarm compared with meteorological conditions (A) Example of a locust swarm (Courtesy: Bilal Tarabey-AFP). (B) Finite element model showing the effect of a locust swarm on atmospheric PG (in V/m). Color scale truncated above 300 V/m. (C) Charge densities of several swarming insect species and meteorological phenomena – from top to bottom, honeybee (*Apis mellifera*), Lepidoptera (*Tyria jacobaeae* and *Aglais io*) (England and Robert, 2022), desert locust (*S. gregaria*), semi-fair-weather clouds, thunderstorm clouds, and electrified dust storms.

Insect swarms can occur at different altitudes and with different densities, and often consist of a single species (e.g., honeybees, locusts, butterflies, see Figures 1A and 2A). Using published charge measurements of honeybees (Edwards, 1962; Erickson, 1975) and our own calibrated measurements, we developed a three-dimensional finite element analysis model to computationally evaluate the effect of swarming honeybees on local atmospheric electric fields. These models of the electric fields around honeybee swarms highlight the appreciable perturbing influence that they can have on their local electrical environment (Figure 1B).

To test whether honeybee swarms can indeed cause observable atmospheric PG variations, measurements were carried out at our field station at the University of Bristol, School of Veterinary Sciences, Langford, United Kingdom. The site contains several honeybee hives used for research, which, in the event of overcrowding, exhibit their typical swarming behavior. Such an event provided an opportunity to assess the electrical effect of a honeybee swarm, by placing an electric field monitor (Boltek EFM 100 Field Mill) and an upward viewing camera near the swarm to assess swarm density. For about three minutes, part of the migrating swarm passed over the electric field monitor (Figure 1A). This revealed a net positive PG increase of 100 Vm^–1^ at peak density (Figure 1C). This effect was not observed in a second control field mill positioned in the open field 50 m away from the swarm (Inset Figure 1C). Cross-correlation indicates that bee swarm density (expressed as relative pixel density) was highly correlated with atmospheric PG (*R*^2^ = 0.92, *p* < 0.001; Figure 1D). Regression analysis suggests a linear correlation between relative swarm density and atmospheric PG (*R*^2^ = 0.85, *p* < 0.001; Figure 1E). Collectively, this evidence suggests that a honeybee swarm contains enough charge to affect the atmospheric PG, proportional to the swarm density. In several other swarming events, net positive increases in atmospheric PG have been observed up to ∼1,000 Vm^–1^ at peak density (Figure 1F). The observed magnitude of the density-dependent effects thus falls within the expected ranges predicted in our model (Figure 1B).

### Relevance of insect swarms for atmospheric electricity

Various insects swarm, including Hymenoptera (e.g., wasps and flying ants), Isoptera (e.g., termites), Orthoptera (e.g., locusts), Lepidoptera (e.g., migrating butterflies and moths), Diptera (e.g., mosquitoes, midges, and cluster flies), Ephemeroptera (e.g., mayflies), and Coleoptera (e.g., Australian soldier beetles). Gregarious grasshoppers, or locusts (Acrididae), can form swarms on a *biblical* scale (covering land areas of up to 1,000 km^2^ (Uvarov, 1977); (Figure 2A), causing severe damage to agriculture and subsistence farming by decimating crops (Zhang et al., 2019). We therefore also estimated the effect of swarming locusts on local atmospheric electric fields based on our measurements of locusts' charges (805 ± 295.5 pC, median ±SD, *N* = 8) and published densities (0.001–10 insects m^–3^; Rainey, 1958). Using a similar rationale to that used here for honeybees, our analysis reveals that locust swarms have the potential to alter their local electrical environment with a magnitude comparable with meteorological events (Figure 2B).

To provide context between insect swarm charges and meteorological phenomena, we estimated charge densities of swarms of three different swarming insects and compared these to meteorological charge densities (semi-fair-weather clouds, storm clouds, and electrified dust storms) reported in the literature (Harrison et al., 2018; Marshall and Stolzenburg, 1998; Nicoll and Harrison, 2016). Our calculations show that desert locust (*Schistocerca gregaria*) swarms are capable of exceeding charge densities reported for electrical storms and clouds (Figure 2C) (Marshall and Stolzenburg, 1998). In contrast, Lepidoptera (moths and butterflies) (England and Robert, 2022) do not appear to represent substantial sources of atmospheric charge when compared with meteorological phenomena owing to their low average densities (∼0.01 insects m^–3^) reported in the literature (Figure 2C; Johnson, 1969). However, extreme swarming incidents have been reported (Callahan and Mankin, 1979), in which it can be estimated that variations in PG resulting from such densities could potentially be commensurate with the PG changes associated with fair-weather clouds.

### Concluding remarks and limitations of the study

The presented evidence that swarming, migrating insects transport charge in the lower atmosphere indicates that large collections of charged insects will contribute to a hitherto unrecognized source of electrical variability in the atmosphere. This recognition potentially carries various physically- and biologically relevant implications. For instance, entomogenic space charge is not considered in current climate models aimed at capturing the complex interplay between radiation and particulate matter, such as the atmospheric transport of dust. As atmospheric space charge enhances the aggregation and movement of aerial particles (Toth et al., 2020), it is conceivable that insect-derived space charges will also contribute to spatial changes in aerial particles. For example, it could be speculated that insect-driven charged particle collection and transport could contribute to long-range transport of desert dust, providing alternative explanations for the transport of large particles, which cannot be explained by physical processes alone (Toth et al., 2020; Does Van der et al., 2018). Further, insects are not the only source of biogenic charge in the atmosphere, as birds and microorganisms also carry charge and abound in the lower atmosphere (Badger et al., 2015; de Groot et al., 2021). The observed presence and magnitude of biogenic space charge invites further interdisciplinary research into the dynamic electrical interactions between physical and biological entities in the atmosphere.

## STAR★Methods

### Key resources table

**Table:** 

REAGENT or RESOURCE	SOURCE	IDENTIFIER
Other	Boltek EFM 100	N/A
Other	AKASO V50×	N/A
Other	John Chubb Instrumentation Ltd. Cheltenham, UK	N/A
Software and Algorithms	COMSOL Multiphysics® v. 5.4 (COMSOL AB, Stockholm, Sweden)	N/A
Depoisted Data	Mendeley Data	https://doi.org/10.17632/wzxpcn9r55.1
Deposited Data	Mendeley Data	https://doi.org/10.17632/5bmscj7jf7.1

### Resource availability

#### Lead contact

Further information and requests for resources and reagents should be directed to and will be fulfilled by the lead contact, Ellard Hunting, e.r.hunting@bristol.ac.uk/e.r.hunting@gmail.com.

#### Materials availability

This study did not generate new unique reagents.

### Method details

#### Finite element analysis

Modelling of electric fields was performed using finite element analysis within COMSOL Multiphysics® v. 5.4 (COMSOL AB, Stockholm, Sweden) utilising the “Electrostatics” interface within the “AC/DC” module. The three-dimensional geometry consisted of a 60 m × 60 m x 40 m (length, width, height) cuboid within which the model operated. The two runs made for differently sized and charged ellipsoids represented insect swarms of honeybees and locusts. The honeybee swarm was represented as an ellipsoid with semi-axes 2 m x 1 m x 0.5 m ellipsoid at a height of 3 m, and the locust swarm an ellipsoid with semi-axes 4 m x 1 m x 1 m at a height of 15 m. The swarm charge was distributed evenly as a volume charge within the ellipsoids, with the total charge calculated respectively as the sum of 500 bees each carrying +100 pC (order of magnitude from (England and Robert, 2022)) and 1000 locusts carrying +850 pC each. An approximately 8 m tall deciduous tree was included in each model to provide scale and an electrical landmark for comparison (Hunting et al., 2021a). The remainder of the model domain was assigned as air. The upper surface of this air column was given an electrical potential typical of a 40 m altitude in fair-weather conditions (+4 kV), with the bottom surface defined as zero potential, equivalent to the established surface (first meter) atmospheric potential gradient of 100 V/m (Bennett and Harrison, 2007; Wilson, 1903). The surface of the tree was also defined as ground (Hunting et al., 2021a). Meshing of the geometry was physics-controlled, set to “extremely fine”. The relative permittivity, ε_r_, was defined as ε_r_ = 12 for living trees (Hunting et al., 2021a; Suojanen et al., 2001), ε_r_ = 1 for air, and ε_r_ = 80 inside the insect swarms (likely an overestimate, based on the permittivity of water). Model outputs presented for this study were produced by plotting data from two-dimensional slices through the centre of the three-dimensional dataset.

#### Measurements of swarms, insect charges, and electric fields

Observations were carried out at our field station at the University of Bristol, School of Veterinary Sciences, Langford United Kingdom, which is equipped with an electric field monitor to continuously measure atmospheric PG (Boltek EFM 100 Field Mill, calibrated in a capacitor plat setup). This site features several honeybee hives that are used for research. In the event of overcrowding of a beehive, the original queen leaves the hive with a fraction of the workers (on average around 12,000 bees) (Fell et al., 1977), resulting in occasional swarming events near PG measuring equipment at our study site. When honeybees swarm, they usually cluster on a limb of a tree for several days while scout bees search for suitable cavities to nest in. After an appropriate nest is found, the swarm will collectively migrate. An electric field monitor was placed near the swarm. A camera (AKASO V50×, 30 fps) was positioned next to the field mill with an upward orientation to record the swarm in flight (Figure 1B). A second electric field monitor was placed in an open field, 50m away from the other electric field monitor and swarm to ascertain any dynamics in the electric field monitor was caused by the presence of a swarm. For about three minutes, part of the swarm passed over the electric field monitor.

### Quantification and statistical analysis

The video recording capturing the swarm event was cropped to a 500 by 220-pixel window to remove foreground objects obstructing the view of the swarm and was analysed using a custom script in Python 3.8.1. The video was converted to black and white pixels such that the background was white and any non-background objects, comprising the swarm, were black. The ratio of black to white pixels was calculated for each frame of the video resulting in a proxy measure for bee density, defined as relative pixel density here. At points during the swarm’s passage, flowering heads of grass entered the frame, increasing the relative pixel density. Such affected data were therefore removed. The relative pixel densities were filtered by a moving mean over 10 data points (corresponding to 0.5 s) to emphasise the long-term trend of the data. These data were compared to data collected by the electric field monitor using cross correlation and linear regression in PAST v.4. Since insect charges can be expected to influence PGs directly, data were aligned (zero lag) based on the highest cross correlation coefficient.

To compare the point charge densities of insect swarms with electrical meteorological phenomena, insect surface charges were combined with published insect swarm densities (Johnson, 1969; Rainey, 1958). Surface charges were either taken from the literature (England and Robert, 2022) or measured within Faraday cages in a laboratory setting. Each individual insect was treated as a point charge and 3-D distribution was assumed to be homogenous. Surface charges of the desert locust (*Schistocerca gregaria*) were measured using a manufacturer-calibrated JCI 147 Faraday pail and a JCI 140 static monitor (John Chubb Instrumentation Ltd. Cheltenham, UK), and those of one butterfly and one moth species (*Aglais io*, Nymphalidae and *Tyria jacobaeae*, Erebidae, respectively) were measured using a calibrated induction ring electrode and custom-built picoammeter. Insects were dropped or flew into the pail or through the induction ring without any contact from the experimenter, the equipment, or electrically charged or conductive objects. As insects accumulate surface charge during flight (e.g., (Erickson, 1975)), we allowed for such charging prior to measurement. Charging either consisted of tethering the insect with fishing line and allowing the animal to fly for a minimum of 30 s or placing the insect in a wind tunnel consisting of a plastic tube with plastic mesh at both ends with a 120 mm USB-powered computer cooling fan at one end. The airflow over the insect simulated flight in terms of friction with air for a triboelectric charging effect, but also through collision with airborne ions for an ion adsorption charging effect (Lighthart et al., 2005).

## Data Availability

• All data have been deposited at Mendeley Data, and are publicly available as of the date of publication. DOI is listed in the key resources table. Any additional information required to reanalyze the data reported in this paper is available from the lead contact upon request. • All codes have been deposited at Mendeley Data, and are publicly available as of the date of publication. DOI is listed in the key resources table. Any additional information required to use the code reported in this paper is available from the lead contact upon request.
